# Development of a User-Friendly Self-Screening Tool for Assessing Metabolic Syndrome Risk in Youths from Economically Challenged Regions

**DOI:** 10.3390/jpm14080810

**Published:** 2024-07-30

**Authors:** Jacqueline Fernandes de Sa Xavier, Shirley C. Feuerstein, Augusto Cesar Ferreira De Moraes, Tiago Almeida de Oliveira, Evellyn Ravena da Silva Gomes, Maria Isabela Alves de Almeida Silva, Luiz Fernando de Oliveira, Heraclito Barbosa de Carvalho, Kliver Antonio Marin, Marcus Vinicius Nascimento-Ferreira

**Affiliations:** 1 Health, Physical Activity and Behavior Research (HEALTHY-BRA) Group, Universidade Federal do Tocantins, Miracema do Tocantins 77650-000, Brazil; jacsafernandes@yahoo.com.br (J.F.d.S.X.); shirleycunha_@hotmail.com (S.C.F.); eviravena90@gmail.com (E.R.d.S.G.); mariaisabelaalmeida15@gmail.com (M.I.A.d.A.S.); oliveira.luiz@mail.uft.edu.br (L.F.d.O.); klivermarin@mail.uft.edu.br (K.A.M.); 2 Texas PARC—Texas Physical Activity Research Collaborative Lab, Michael and Susan Dell Center for Healthy Living, Department of Epidemiology, School of Public Health in Austin, The University of Texas Health Science Center at Houston, Austin, TX 78701, USA; augusto.cesar.ferreirademoraes@uth.tmc.edu; 3 Statistic Department, State University of Paraiba, Campina Grande 58429-900, Brazil; tadolive@servidor.uepb.edu.br; 4 Instituto de Ensino Superior do Sul do Maranhão (IESMA/UNISULMA), Imperatriz 65907-070, Brazil; 5 YCARE (Youth/Child and Cardiovascular Risk and Environmental) Research Group, School of Medicine, University of Sao Paulo, São Paulo 17012-900, Brazil; heracc@usp.br

**Keywords:** nomogram, disparities, lifestyle, metabolic health

## Abstract

Background: Metabolic syndrome increases the risk of heart disease and diabetes. Early identification and management are crucial, especially in economically challenged regions with limited healthcare access. Aims: To develop nomograms for individualized risk estimation for metabolic syndrome in young people from low-income regions. Methods: We assessed 496 college students from two Brazilian cities with Gini indices ≤0.56. Of these, 69.9% were female, 65.1% were younger than 20 years, 71.8% were non-white, and 64.3% were enrolled in health-related courses. For external validity, we assessed metabolic syndrome in a subset of 375 students. Results: We found 10 variables associated with abdominal obesity by logistic regression: age, biological sex, physical education facilities, enrollment in sports competitions during elementary school, grade retention, physical education as the preferred subject, physical education classes per week, and enrollment in sports training in secondary school (score A); adherence to 24 h movement behaviors (B score); and body weight (score C). We designed three nomograms (for scores A, B, and C), all of which showed acceptable performance according to the area under the receiver operating characteristic curve (≥0.70) and calibration (Hosmer–Lemeshow test, *p* > 0.05). In the external validation, we observed higher predictive capability for the A and B scores, while the C score had lower but still acceptable predictive ability. Conclusions: User-friendly self-reported data accurately predict metabolic syndrome among youths from economically challenging areas.

## 1. Introduction

Metabolic syndrome is a clinical condition characterized by the simultaneous presence of several metabolic disorders, such as abdominal obesity, arterial hypertension, hypertriglyceridemia, and hyperglycemia [1]. This condition has a high prevalence worldwide of between 3.0% and 5.0% in children and adolescents, with rates often higher in low-income regions [2]. Obesity has emerged as the main risk factor for the development of metabolic syndrome [3], remaining throughout life [4], with more than half of the pediatric population (57.3%) becoming obese before 35 years of age [4]. Additionally, adults who are overweight or obese are 5 times more likely to have metabolic syndrome [5].

Thus, young people are priority candidates for early interventions. However, combating metabolic syndrome in this population is a challenge. The most convincing reason may be the complex processes underlying the etiology of metabolic syndrome, in which any single predictor is unlikely to have a dramatic impact or to fully explain the risk of the outcome [1]. Given this complexity, it is necessary to construct a robust predictive model that incorporates multiple established risk factors and considers the social disparities involved [6]. Many studies have sought the risk factors for metabolic syndrome in young adults [3,5,7,8]. Interpreting and understanding the results is a challenge in medical practice and for individuals with limited statistical skills [9]. To overcome this limitation [10], nomograms have been frequently used in the medical literature on metabolic health [9,11,12,13,14,15,16,17].

A nomogram assists clinical decision-making via a user-friendly graphical interface, generating an individualized risk estimate of the outcome based on patient characteristics [10]. Nomograms have been made to predict abdominal obesity [11] and metabolic syndrome [9,12]. These models are centered on measures such as waist circumference, fat percentage, blood pressure, and family history. No models are available or even reproducible for young people that are based on a combination of demographic, economic, and contextual factors [18]. These issues are shaped by social disparities in health [19], with modifiable behaviors [20], accessibility, and low cost. In addition, few models are sensitive to socioeconomic gradients [18]. Predictors that are not dependent on the controlled or clinical environment are applicable to low-income regions. Models adapted to triage individuals with a high probability of having limited access to healthcare, who often have a late diagnosis and no treatment for metabolic syndrome [18] due to social disparities, are needed. Thus, the objective of this study was to develop and validate nomograms for the individualized estimation of the risk of metabolic syndrome in young people from low-income regions.

## 2. Materials and Methods

### 2.1. Study Design

**Figure 1:**
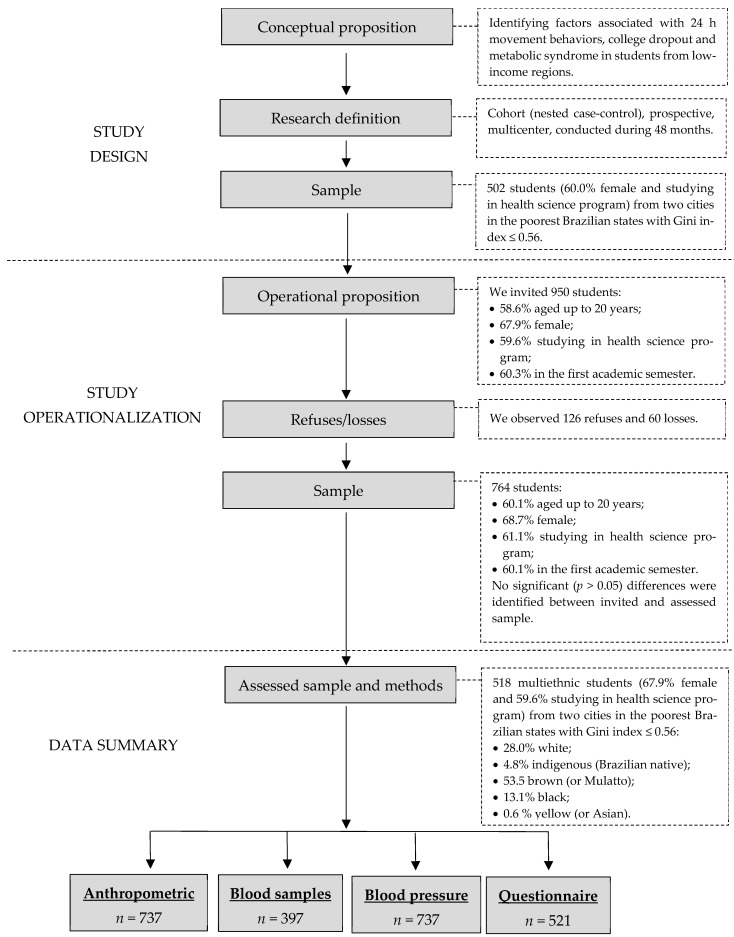
Sample and cohort study design.

This study is part of the multicenter longitudinal project 24 h movement behavior and metabolic syndrome (24 h-MESYN) [21] and was conducted with baseline data. This project was designed to study the intersectionality of health determinants [19], considering different levels of social disparities structured in low-income regions: macro (economic (Gini indices) and political (states and cities)), meso (public and private institutions), and micro (biological sex, race/ethnicity and economic status)). Figure 1 shows the design of the 24 h-MESYN, the operationalization of the baseline data, and the summarized data accessed. The detailed method of the 24 h-MESYN project is available in the literature [21].

### 2.2. Ethical Aspects

The 24-MESYN project was reviewed and approved by the Research Ethics Committee (CEP, Nos. 4055604 and 5161340) and followed national (e.g., CNS Resolution 466/12) and international standards (e.g., Declaration of Helsinki). The students or their guardians reviewed and signed the informed consent form and/or assent form prior to participation [21].

### 2.3. Participants

The students were recruited from two research centers, a private university in Imperatriz, Maranhão, Brazil, and a public university in the city of Miracema do Tocantins, Tocantins, Brazil. Together, these research centers reported 3200 students in 2021. Both cities are in the poorest states of the country, with a Gini index of 0.56 [22] for Imperatriz and 0.43 [23] for Miracema do Tocantins. Of the 950 students invited, 186 refused to participate or were considered to have missing data. The final sample included 794 participants, 521 of whom responded to the questionnaires. We did not observe statistically significant differences in the distributions of students between the included and no questionnaire provided (Figure 1).

Thus, in the present study, we included 521 students aged 17 years or older who were enrolled in the institutions of this project, were in the first year of their undergraduate course, and submitted the informed consent form and/or assent form. We excluded 25 students with incomplete data about school context or 24-h movement behavior. The final study sample included 496 students, of whom 69.9% were female and 30.1% male, 65.1% were younger than 20 years of age, 71.8% reported being of non-white race/ethnicity (including 4.8% Indigenous, 53.6% mixed race, and 13.4% black or Asian), and 64.3% were enrolled in courses in health-related fields. A subsample of 397 students volunteered for blood collection, of whom 375 had complete information (questionnaire, anthropometry, and blood sample; Figure 1).

### 2.4. Variables

We assessed demographic, economic, and educational characteristics, 24 h movement behaviors, and body weight as potential predictors. We considered abdominal obesity and metabolic syndrome as outcomes. We operationalized the potential predictors using a self-reported online questionnaire (available at https://forms.gle/zgjAKCAArngqXxtz6, accessed on 29 July 2024) [21], while outcomes (and body weight) were assessed objectively.

#### 2.4.1. Demographic and Economic Variables

We evaluated the demographic predictors of age (converted into age group (≤18 years; 19–20 years, or ≥21 years)), biological sex (male or female), and race/ethnicity (white, black, brown or mulatto, Indigenous, or oriental or Asian). Regarding the economic aspects, we retrieved information on the students’ profession (converted into working student (yes or no)) and monthly family income in Brazilian reais (R$, converted into the minimum wage range, R$1320.00 (US$260.15) into 2023 Brazilian reais) [24].

#### 2.4.2. School Context

We assessed school predictors at the elementary and high school levels. The students reported the following: grade retention (any failures or repeats (yes or no), physical education facilities (availability of sports courts/fields for physical education (yes or no)), preferred curricular component (Arts, Sciences, Physical Education, Religious Education, Geography, History, English, Portuguese, or Mathematics; converted to Physical Education as preferred subject (yes or no)), physical education classes per week (no classes, 1 class per week, 2 classes per week, 3 classes per week, 4 classes per week, 5 classes per week, or more than five classes per week), enrollment in sports training (participation in competitive school training (no enrollment, once a week, twice a week, three times a week, four times a week, five times per week, six times per week, or seven times per week)), and enrollment in sports training (participation in competitive school sports (yes or no)).

#### 2.4.3. 24 h Movement Behaviors

Twenty-four-hour movement behaviors were assessed via 19 items addressing the duration of physical activity, sedentary behavior, and daily sleep [25]. We assessed physical activity using the International Physical Activity Questionnaire (IPAQ) short version [26,27]. This instrument assesses physical activity using six items on the frequency, duration, and intensity of activities (light (walking), moderate, and vigorous) [27]. Sedentary behavior was assessed using the South American Youth Cardiovascular and Environmental (SAYCARE) questionnaire [28,29]. The instrument consisted of 10 questions about the time spent on weekdays and weekends in activities such as watching television, using a computer or cell phone, studying, reading books or magazines, playing electronic games, and passive commuting [29]. We examined sleep quality over time with the Pittsburgh Sleep Quality Index (PSQI), a questionnaire that assesses sleep quality over a 1-month period [30]. This questionnaire comprises 19 items that assess seven sleep components (sleep quality, sleep latency, sleep duration, habitual sleep economy, sleep disorders, sleep disorders, use of sleep medications, and sleep dysfunction) [31]. In this study, we used only three items from the sleep duration dimension [31]. In a pilot study with 195 participants [21], we tested the psychometric properties of these items applied online and found acceptable test–retest reliability (2-week interval; correlation coefficient > 0.30) and structural validity (exploratory factor analysis; loading factor > 0.30).

We classified 24 h movement behavior by whether it adhered to international recommendations [25,32]. The adopted criteria were as follows: at least 60 min of physical activity daily until the age of 17 years and at least 30 min daily for those aged 18 years or older; sedentary behavior, limited to a maximum of 2 h a day until the age of 17 years and 8 h a day for those aged 18 years or older; and sleeping 8 to 10 h per night until the age of 17 years and 7 to 9 h per night for those aged 18 years or older. We identified each participant based on the extent to which they achieved recommendations for 24 h movement behaviors (0, 1, 2, or 3).

#### 2.4.4. Body Weight

We performed the measurements in a private room at the participant’s institution. All measurements were performed with participants wearing as light clothing as possible and no footwear. Next, we asked the participants to step on the digital scale (Omron Healthcare, Kyoto, Japan) for weight measurement. We performed two measurements, with a third measurement being taken if the difference between the first two exceeded the allowable tolerance (100 g) [21]. The individuals stood upright looking straight ahead during the measurements [21].

#### 2.4.5. Abdominal Obesity

We operationalized abdominal obesity using waist circumference, according to international standards [33]. The waist circumference was measured twice with inelastic tape at the midpoint between the lower edge of the last rib and the upper iliac crest [34]. A third measurement was taken if the difference between the first and second measurements was greater than 5.0% [21]. The evaluations were performed in a private room at the institution. All measurements were performed with participants wearing light clothing and no footwear [21]. We considered waist circumferences ≥90 cm in men and ≥80 cm in women abnormal [1].

#### 2.4.6. Metabolic Syndrome

Metabolic syndrome was diagnosed based on the criteria of the International Diabetes Federation [1], which defines it as the presence of three of five risk factors: high abdominal circumference (≥90 cm in men; ≥80 cm in women), high blood pressure (systolic ≥ 130 and/or diastolic ≥ 85 mmHg), high triglycerides (TG, ≥150 mg/dL), low HDL-cholesterol (HDL-c, ≤40 mg/dL in men; ≤50 mg/dL in women), and high fasting blood glucose (≥100 mg/dL). Blood pressure measurements were performed according to standardized recommendations [35]. We performed the measurements with the Omron automatic arm oscillometric device HEM-7320-LA (Omron Health care, Kyoto, Japan), with a pressure range of 0–299 mmHg and a heart rate range of 40–180 beats/minute [21]. Blood pressure and heart rate were measured twice 2 min apart, according to international guidelines [36]. If the values of the second measurement were more than 5.0% away from the first, a third measurement was taken [36].

A venous blood sample was collected using a Vacutainer system (Becton Dickinson, Oxford, UK). This procedure was performed in the morning after an overnight fast of 8–12 h [21]. We instructed participants to fast on the morning of the collection day. The samples were collected and analyzed by a previously accredited laboratory following international protocols and recommendations [37]. These included not performing moderate to vigorous physical activity and not consuming products with caffeine or alcoholic beverages, in addition to avoiding drinking water in the hours before the evaluations.

### 2.5. Procedures

To harmonize the measures, the researchers underwent 60 h of fieldwork, distributed in three 20 h training sessions, in 2021 (before the pilot study), 2022, and 2023 (before the baseline data collection). The training programs included unifying the protocol for inviting participants, distribution and receipt of online questionnaires, and data collection using objective instruments. In the first stage of the research procedure, we conducted an invitation, an explanation of the project, and delivery of the informed consent form and/or assent form. In the second stage, after returning the signed form, the participants underwent an anthropometric evaluation. In the third stage, they answered the online questionnaires. In the fourth stage, a blood sample was drawn [21].

### 2.6. Statistical Analysis

Our analyses were performed using Stata software, version 16.0 (Stata Corporation, College Station, TX, USA). We considered *p* < 0.05 statistically significant. We conducted a descriptive analysis stratified by the presence of abdominal obesity, and the results are presented as the frequency (%). We examined the bivariate differences between these variables with the chi-squared test.

The modeling followed the construction, discrimination, calibration, and validation protocol for nomograms [10]. For the selection of predictors, we constructed multilevel logistic regression models adopting abdominal obesity as the primary outcome measure. The random-effects models were adjusted to analyze the relationships with the potential predictors at three levels of data organization: distal (demographic and economic factors), medial (educational factors), and proximal (behavioral factors and body weight). For a variable to be kept in the multivariate model, we set the significance level at *p* ≤ 0.20 [38]. We evaluated the discriminative ability of the predicted probabilities and the observed frequencies via the area under the receiver operating characteristic curve (AUROC), with the acceptable predictive ability being defined as AUROC ≥ 0.70 [39]. We evaluated the calibration of the models fitted with the Hosmer–Lemeshow test, where *p* > 0.05 indicated an acceptable fit. We tested the internal validity of the scores using bootstrapping (B = 150) [10]. For external validity, we compared the discriminative ability of the scores for the incidence of metabolic syndrome (secondary outcome). We present the predictive models (nomograms) graphically, along with the equations (multivariate logistic regression coefficients) used to generate them.

## 3. Results

Among the 496 students evaluated, 23.0% had abdominal obesity. We identified statistically significant differences in the distribution of the primary outcome by age (*p* < 0.001), biological sex (*p* = 0.031), working student (*p* = 0.012), monthly household income (*p* = 0.025), availability of physical education facilities (*p* = 0.009), physical education classes per week (*p* = 0.043) in elementary school, grade retention (*p* = 0.014) in high school, and 24 h movement behavior score (*p* = 0.011). The demographic, economic, behavioral, and metabolic characteristics of the students are presented in Table 1, while Table 2 presents the school variables.

Table 3 presents the construction of the scores and their respective predictive capabilities and calibrations. The following potential predictors were included in the multilevel logistic regression models (*p* ≤ 0.20): age, biological sex, physical structure for physical education classes and enrollment in sports competitions (during elementary school), grade retention, physical education as preferred subject, physical education classes per week and enrollment in sports training (during high school), adherence to 24 h movement behaviors recommendations, and body weight. The retained factors are divided into three models, with gradual adjustment (deviance) to the cumulative level of information. The models showed acceptable predictive capabilities, with the following AUROCs: score A, 0.70 (95% CI: 0.64–0.75); score B, 0.70 (95% CI: 0.65–0.76); and score C, 0.94 (95% CI: 0.92–0.96). All scores had acceptable calibration (*p* > 0.05). The nomogram equations based on the retained predictors are as follows:

$$\begin{array}{c} Score\;A=100\%\;\times 1/\{1+\exp[-(-3.12+0.58\times \mathrm{age}\;+0.59\times \mathrm{biol}\_\mathrm{sex}\;+0.38\times \mathrm{PEfacilityES}\\ +0.53\times \mathrm{sport}\_\mathrm{comp}\_\mathrm{ES}\;+0.47\times \mathrm{grade}\_\mathrm{ret}\_\mathrm{HS}\;+0.18*\mathrm{PE}\_\mathrm{pref}\_\mathrm{HS}\\ +0.23\times \mathrm{PEclass}\_\mathrm{HS}\;+0.28\times \mathrm{sports}\_\mathrm{train}\_\mathrm{HS})]\} \end{array}$$

$$\begin{array}{c} Score\;B=100\%\;\times 1/\{1+\exp[-(-2.81+0.59\times \mathrm{age}\;+0.54\times \mathrm{biol}\_\mathrm{sex}\;+0.41\times \mathrm{PEfacilityES}\;\\ +0.53\times \mathrm{sport}\_\mathrm{comp}\_\mathrm{ES}\;+0.46\times \mathrm{grade}\_\mathrm{ret}\_\mathrm{HS}\;+0.20\times \mathrm{PE}\_\mathrm{pref}\_\mathrm{HS}\;+0.23\times \mathrm{PEclass}\_\mathrm{HS}\;\\ +0.27\times \mathrm{sports}\_\mathrm{train}\_\mathrm{HS}\;+-0.19\times \mathrm{score}24h)]\} \end{array}$$

$$\begin{array}{c} Score\;C=100\%\;\times 1/\{1+\exp[-(-9.73+0.36\times \mathrm{age}\;+3.11\times \mathrm{biol}\_\mathrm{sex}\;+0.58\times \mathrm{PEfacilityES}\;\\ +0.79\times \mathrm{sport}\_\mathrm{comp}\_\mathrm{ES}\;+0.53\times \mathrm{grade}\_\mathrm{ret}\_\mathrm{HS}\;+0.26\times \mathrm{PE}\_\mathrm{pref}\_\mathrm{HS}\;+0.26\times \mathrm{PEclass}\_\mathrm{HS}\;\\ +0.27\times \mathrm{sports}\_\mathrm{train}\_\mathrm{HS}\;+-0.25\times \mathrm{score}24h\;+2.70\times \mathrm{weight})]\} \end{array}$$

**Figure 2:**
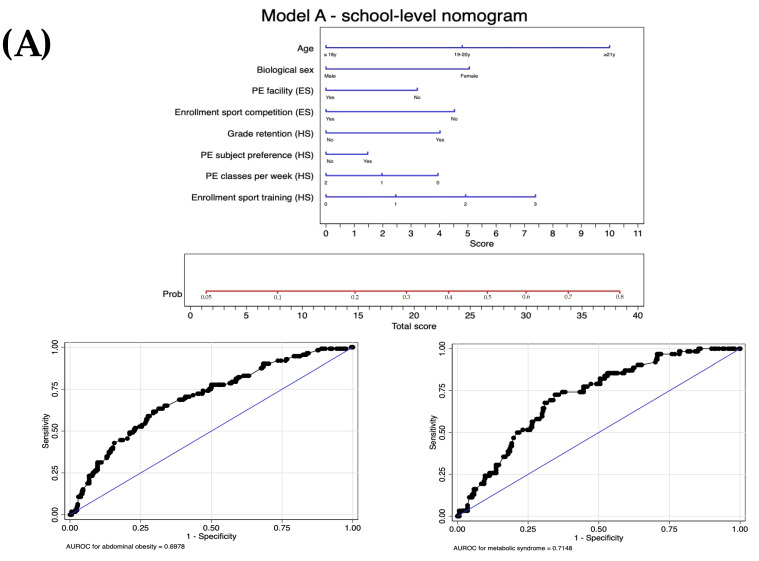

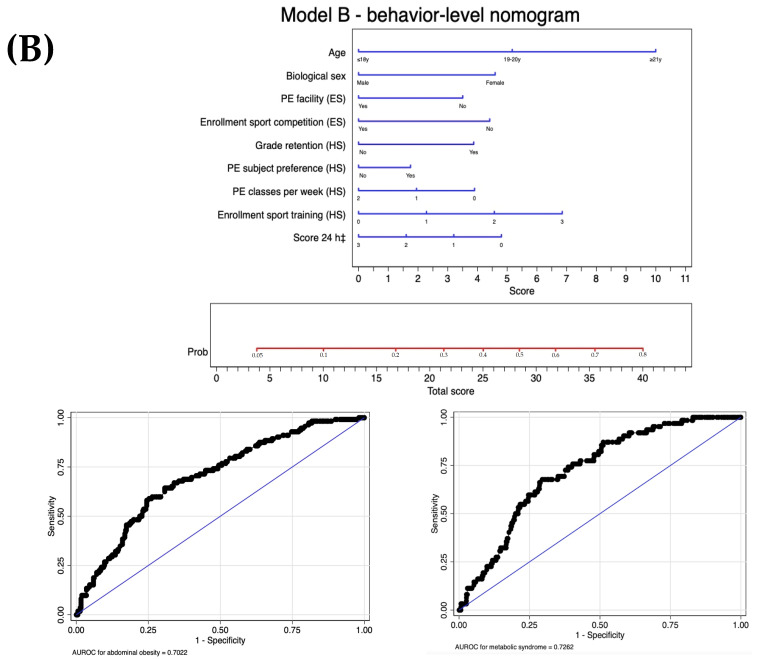

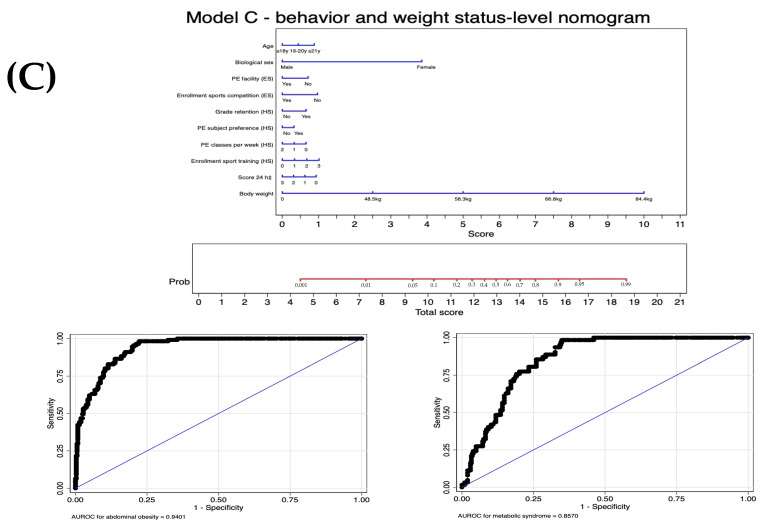
Nomogram and area under the receiver-operating characteristic curve (AUROC) for abdominal obesity and metabolic syndrome predicting. School-level model (**A**), behavior-level model (**B**), and behavior- and weight status-level model (**C**). ES, elementary school; HS, high school; PE, physical education. ^‡^ Sum of the 3 variables (physical activity + screen time + sleep time) to estimate the degree of 24 h movement behaviors (0, 1, 2, or 3) recommendations are accomplished.

In the external validation, we observed an increase in the predictive capacity for metabolic syndrome of score A (AUROC = 0.72 [95% CI: 0.63–0.74]) and score B (AUROC = 0.73 [95% CI: 0.63–0.76]), while there was a decrease in the predictive performance of score C (AUROC = 0.86 [95% CI: 0.81–0.88]). We graphically present the nomograms and their corresponding predictive capabilities for abdominal obesity and metabolic syndrome separately in Figure 2.

## 4. Discussion

The novelty of this study was the development of low-cost, simple, and rapid predictive nomograms to estimate the risk of metabolic syndrome in low-income regions. Unlike previous studies, all of the predictors included in our models can be obtained by the participants themselves, making the 24-h MESYN risk scores easy to use. Our nomograms included predictors such as age, biological sex, facilities for physical education classes and enrollment in sports competitions (during elementary school), grade retention, physical education as a preferred subject, physical education classes per week and enrollment in sports training (during high school), adherence to movement behaviors within 24 h, and body weight. The 24 h-MESYN risk scores can predict an individual’s risk of a metabolic outcome [1] by integrating several prognostic variables [9,11,13,14,15,16,17] and determinants [5,6,7,40] reported in the literature; however, it also meets the need for simplified screening models that include school predictors [41,42,43] fulfilling a dual function, namely, use in personalized medicine and self-assessment in young people from economically disadvantaged regions.

Metabolic syndrome is a multifactorial outcome [1]. Along with demographic and economic factors (e.g., biological sex and family income) [9], lifestyle plays an important role in adults, including young people [5,7]. The literature underscores obesity, particularly abdominal obesity, as a primary factor in children and adolescents [3,8], contributing significantly to metabolic syndrome [44]. The pathophysiology involves complex mechanisms that remain incompletely understood [45], with insulin resistance, chronic inflammation, and neurohormonal activation emerging as pivotal in its progression toward cardiovascular disease and type 2 diabetes [44,45]. During early life, obesity is influenced not only by 24 h movement behaviors [20,42] but also by school-related factors such as grade retention, physical education classes, and sports activities [41,42]. From this perspective, we hierarchically organized the potential predictors that were consistently associated with the risk of obesity during schooling and metabolic health in adults into three levels (educational, behavioral, and body weight), in line with previous studies [5,6,7,9,11,13,14,15,16,17,20,40,41,42]. We believe that this process of constructing our models partially explains their predictive performance, with the third-level score (24 h-MESYN score C) having a similar or greater predictive capacity than other, more complex and invasive nomograms for abdominal obesity [11] and metabolic syndrome [9,12].

The 24 h-MESYN A and B scores demonstrated acceptable predictive capacity and can be applied, at least for public health screening purposes, in the prevention and management of metabolic syndrome due to their simplicity. To the best of our knowledge, there is no nomogram in the literature for this outcome based only on school context variables [41,42] and/or 24-h movement behaviors [6,7,20], although these factors have been reported to be associated with obesity and metabolic health. However, a score addressing variables such as biological sex, age range, maternal body mass index, active commuting, and consumption of sugar-sweetened beverages indicated a lower-than-acceptable predictive capacity in the school population [11]. Considering these previous findings in the literature, the 24-h MESYN A score emerges as a tool that could be adopted in short-term strategies by aiding decision-making in management programs at the college level, particularly in relation to students’ physical education and sports background. Additionally, it can support long-term strategies by promoting awareness of the school context as an inducer of a healthy profile in underserved communities. Specifically, for the 24 h-MESYN B score, the inclusion of 24 h movement behaviors resulted in a marginal increase in predictive capacity and model fit. We chose to keep these variables, given that the 24-h MESYN is also an educational tool for self-assessment of modifiable behaviors. In addition, to help with lifestyle monitoring and expand its applicability in personalized clinical practice, the tool fulfills a health literacy function, which is especially important for promoting a healthy lifestyle in economically disadvantaged populations [46].

Among all the proposed models, the 24 h-MESYN C score showed the highest predictive ability after the inclusion of body weight as a predictor. The predictive capacity of anthropometric measurements is widely used in the assessment of metabolic health risk [9,11,12,13,14], with body weight [11] being used directly or indirectly via body mass index [9,13,14]. Thus, this score can distinguish young people from low-income regions with metabolic syndrome from those without metabolic syndrome 86.0% of the time. From our point of view, this technique is a promising clinical tool for the identification and diagnosis of metabolic syndrome that is comparable to tools applied in high-income countries [9,12].

Some limitations of this study should be considered. One limitation is the reliance on self-reported data, which may introduce recall bias and social desirability bias [47]. Potentially, the variability in individual interpretations of contexts and behaviors can introduce inconsistencies in the data collected. However, we based the scores on specific environments or behaviors of the school context (e.g., “Indicate how many physical education classes during regular school hours you had on average, per week, during elementary school. Do not count as classes sports activities for competition”) and not on constructs, as indicated in the literature [48], and on validated questionnaires for 24 h movement behaviors. With regard to our sample, it is recommended that at least 10 events be collected for each possible predictor evaluated in the multivariate regression model [49]; we identified 114 (23.0%) students exhibiting the primary outcome. Moreover, although the proposed scores are intended solely for screening and self-care rather than diagnostic purposes, they effectively highlight individual risks for metabolic syndrome, a clinically validated outcome [1]. Finally, our study is based on cross-sectional data so we cannot infer causality [47]. In addition, predictive modeling based on nomograms assumes that the outcome is constant over time [10]. Thus, these nomograms should be validated in studies with similar populations to confirm their clinical utility [10].

The main strength of the 24-h MESYN risk tool, in addition to being simple and rapid, is that its scores are constructed considering established low-cost factors that increase the probability of identifying metabolic syndrome in young people from economically disadvantaged regions, with great potential for reproducibility. We should strive to constantly assess the accuracy and ensure that risk models are both applicable and intuitive at the individual level in this population. Upcoming investigations could explore the ability of the 24-h MESYN risk scores to not only predict outcomes but also to intervene and change those outcomes through health literacy. Future research is needed to develop better strategies for communicating risk in personalized medicine in a way that enhances comprehension. It is also crucial to identify clinically meaningful thresholds and probabilities to implement clinical action items, such as telemonitoring, promoting healthy lifestyles, and body weight monitoring.

## 5. Conclusions

The 24-h MESYN risk scores are valid and accurate tools at three cumulative data levels for the individualized prediction of metabolic syndrome among young people from economically disadvantaged regions. These scores are simple, visual, and easy to use, making them suitable for self-assessment or even public health triage. They may also be helpful in personalized clinical practice for the formulation of strategies and interventions aimed at a healthy lifestyle and body weight, especially when data on metabolic syndrome are unavailable.

## Figures and Tables

**Table 1:** Characteristics of study participants (*n* = 496) based on demographics, economic, behavioral, and metabolic variables.

Variables	Abdominal Obesity		
	No (%)	Yes (%)	*p* Value
Age			
≤18 years	86.4	13.6	<0.001
19–20 years	79.2	20.8	
≥21 years	65.9	34.1	
Biological Sex			
Female	74.4	25.6	0.031
Male	83.2	16.8	
Race/Ethnicity			
White	78.4	21.6	0.371
Indigenous (Brazilian native)	62.5	37.5	
Brown (or Mulatto)	77.7	22.3	
Black and Yellow (or Asian)	75.8	24.2	
Working student			
No	80.4	19.6	0.012
Yes	70.5	29.6	
Monthly household income, R$ *			
<1 minimum wage	82.2	17.8	0.025
1–2 minimum wages	69.9	30.0	
≥3 minimum wages	67.4	32.6	
Score 24 h ^‡^			
0 Meeting 24-h movement	85.0	15.0	0.011
1 Meeting 24-h movement	71.2	28.8	
2 Meeting 24-h movement	76.2	23.8	
3 Meeting 24-h movement	89.7	10.3	
Metabolic syndrome			
No	83.7	16.3	<0.001
Yes	0.0	100.0	

R$, Brazilian (Real) currency; *n*, observations; %, proportion. * Minimum wage in Brazil in May 2023, R$1320.00 (US$260.15). ^‡^ Sum of the 3 variables (physical activity + screen time + sleep time) to estimate the degree of 24 h movement behaviors (0, 1, 2, or 3) recommendations are accomplished.

**Table 2:** Characteristics of study participants (*n* = 496) based on elementary and high school variables.

**Elementary School—Variables**	**Abdominal Obesity**		***p* Value **
	**No (%)**	**Yes (%)**	
Grade retention			
No	78.2	21.8	0.240
Yes	71.8	28.2	
PE facilities			
No	68.3	31.7	0.009
Yes	70.8	20.2	
PE preferred subject			
No	77.0	23.0	0.879
Yes	77.8	22.2	
PE classes per week			
No classes	61.5	38.5	0.043
1 class	77.2	22.8	
≥2 classes	79.9	20.1	
Enrollment in sports training (per week)			
No enrollment	77.9	22.1	
1 session	68.1	31.9	0.121
2 sessions	76.5	23.5	
≥3 sessions	85.7	14.3	
Enrollment in sports competition			
No	73.2	26.8	0.077
Yes	79.9	20.1	
**High school—variables**	**Abdominal obesity**		***p* value **
	**No (%)**	**Yes (%)**	
Grade retention			
No	78.8	21.2	0.014
Yes	64.4	35.6	
PE facilities			
No	70.0	30.0	0.063
Yes	78.8	21.2	
PE preferred subject			
No	77.3	22.7	0.758
Yes	75.4	24.6	
PE classes per week			
No classes	70.8	29.2	0.410
1 class	77.4	22.6	
≥2 classes	78.8	21.2	
Enrollment in sports training (per week)			
No enrollment	79.9	20.1	0.160
1 session	67.2	32.8	
2 sessions	73.8	26.2	
≥3 sessions	75.0	25.0	
Enrollment in sports competition			
No	78.4	21.6	0.414
Yes	75.2	24.8	

PE, physical education; R$, Brazilian (Real) currency; *n*, observations; %, proportion.

**Table 3:** Results of full model to predict abdominal obesity (abnormal waist circumference).

Predictors	Model	Potential Equation *	*p* Value	Deviance	Hosmer-Lemeshow Test	AUROC (95% CI)
Distal: sociodemographic and economic	Level 1	Model 1: Model 0 ^†^		−249.7		
		+Age	0.031			
		+Biological sex	<0.001			
		+Race/Ethnicity	0.477			
	Level 2	Model 2: Model 1				
		+Working student	0.400			
		+Monthly household income	0.827			
Mesial: Elementary school	Level 1	Model 3: Model 2		−173.4		
		+Grade retention	0.926			
		+PE facilities	0.038			
	Level 2	Model 4: Model 3		−241.4		
		+PE as preferred subject	0.744			
		+PE classes per week	0.442			
		+Enrollment in sports training	0.369			
		+Enrollment in sport competition	0.148			
Mesial: High school	Level 1	Model 5: Model 4		−241.6		
		+Grade retention	0.133			
		+PE facility	0.558			
	Level 2: Screening tool A	Model A: Model 5		−240.3	0.46	0.70 (0.64 to 0.75)
		+PE as preferred subject	0.673			
		+PE classes per week	0.176			
		+Enrollment in sports training	0.115			
		+Enrollment in sports competition	0.430			
Proximal: behaviors	Level 1: Screening tool B	Model B: Model A		−239.1	0.77	0.70 (0.65 to 0.76)
		+Score 24 h ^‡^	0.164			
Proximal: behaviors and weight	Level 2: Screening tool C	Model C: Model B		−124.0	0.93	0.94 (0.92 to 0.96)
		+Body weight	<0.001			

CI, confidence interval; *p*: *p*-value; PE, physical education; AUROC, area under the receiver operating characteristics curve. * abdominal obesity as outcome. ^†^ null model. ^‡^ Sum of the 3 variables (physical activity + screen time + sleep time) to estimate the degree of 24 h movement behaviors (0, 1, 2, or 3) recommendations are accomplished.

## Data Availability

The data may be made available upon reasonable request to the researchers.
